# Considerations for Fitting Cochlear Implants Bimodally and to the Single-Sided Deaf

**DOI:** 10.1177/23312165221108259

**Published:** 2022-06-20

**Authors:** Sabrina H. Pieper, Noura Hamze, Stefan Brill, Sabine Hochmuth, Mats Exter, Marek Polak, Andreas Radeloff, Michael Buschermöhle, Mathias Dietz

**Affiliations:** 1 Department of Medical Physics and Acoustic, University of Oldenburg, Oldenburg, Germany; 2 Cluster of Excellence Hearing4all, University of Oldenburg, Oldenburg, Germany; 3 MED-EL Medical Electronics GmbH, Innsbruck, Austria; 4 MED-EL Medical Electronics Germany GmbH, Starnberg, Germany; 5 Division of Otorhinolaryngology, University of Oldenburg, Oldenburg, Germany; 6 Hörzentrum Oldenburg gGmbH, Oldenburg, Germany; 7 Research Center Neurosensory Science, University of Oldenburg, Oldenburg, Germany; 8 KIZMO GmbH, Oldenburg, Germany

**Keywords:** interaural mismatch, loudness balancing, tonotopic mismatch, lateralization bias, binaural fusion

## Abstract

When listening with a cochlear implant through one ear and acoustically through the other, binaural benefits and spatial hearing abilities are generally poorer than in other bilaterally stimulated configurations. With the working hypothesis that binaural neurons require interaurally matched inputs, we review causes for mismatch, their perceptual consequences, and experimental methods for mismatch measurements. The focus is on the three primary interaural dimensions of latency, frequency, and level. Often, the mismatch is not constant, but rather highly stimulus-dependent. We report on mismatch compensation strategies, taking into consideration the specific needs of the respective patient groups. Practical challenges typically faced by audiologists in the proposed fitting procedure are discussed. While improvement in certain areas (e.g., speaker localization) is definitely achievable, a more comprehensive mismatch compensation is a very ambitious endeavor. Even in the hypothetical ideal fitting case, performance is not expected to exceed that of a good bilateral cochlear implant user.

## Introduction

**Figure 1.**
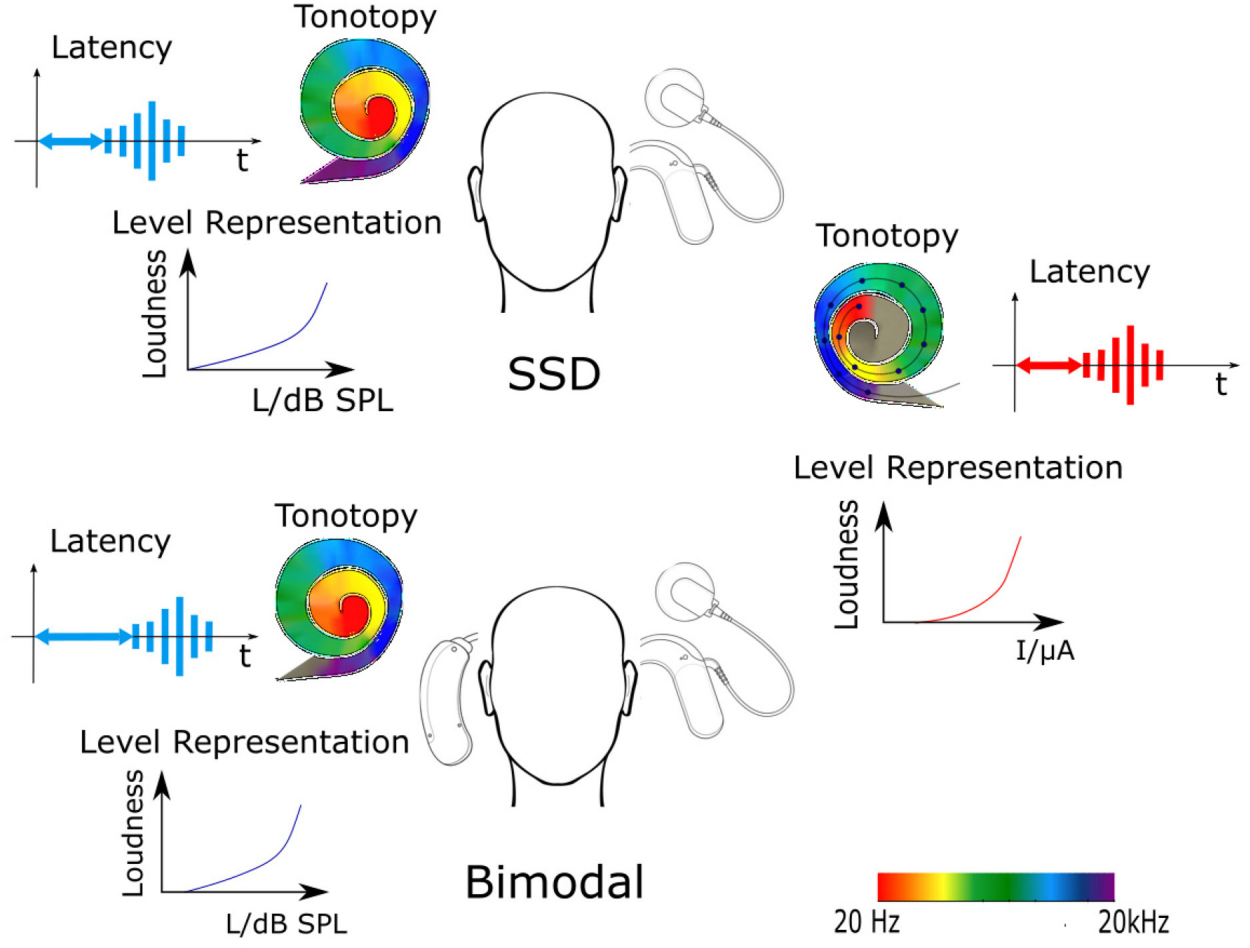
Listening with one electrical and one acoustic ear can lead to different latency, tonotopy, and level representations between the modalities.

Among patients with asymmetric hearing, the largest interaural mismatches exist with one cochlear implant (CI) and acoustic hearing in the other ear (Figure 1). The acoustic hearing can be anything from severely impaired to normal hearing. If the acoustic hearing is supported by a hearing aid (HA), it is referred to as “bimodal CI” and if the acoustic ear is normal hearing, as “single-side deaf CI” (SSD-CI). Average bimodal profiles differ greatly among countries. In the case of restrictive CI funding, patients with one deaf ear and one mild or moderately impaired ear may not receive a CI at all. Under such circumstances, those patients who are provided with a CI are severely impaired in their acoustic ear and their speech perception relies almost entirely on their implanted side with the acoustic side mostly complementing the CI ear with low frequency information. If CI funding is not so restrictive, bimodal users may have two very different, but overall similarly performing, sides or they may have better hearing through the acoustic ear. Especially for these latter patients, a coordinated binaural fitting is expected to be important and these patients are the focus of the present perspective article.

Two primary benefits can arise from listening with two ears: (1) Spatial release from masking, and (2) azimuthal sound localization and the related spatial perception of a sound field. Both benefits are based on the directionally dependent interaural time differences (ITDs) and interaural level differences (ILDs). The ILDs arises from a frequency-depended head shadow and their primary benefit can be understood acoustically, i.e. without considering ear or brain mechanisms. Different ILDs for sounds from different directions often provide a better signal-to-noise ratio (SNR) at one of the two ears, translating into an appropriate masking release, called the head-shadow effect. At high frequencies ILDs can be as large as 20 dB, constituting a potent cue for deciding whether the sound source is on the left or on the right (Blauert, 1997). The benefits are robust and also available to subjects with various types of hearing impairment and of hearing devices (Gifford et al., 2014), as long as they have level-sensitive sound perception in both ears. In addition to the head-shadow effect, binaural neurons in the brainstem can exploit interaural differences using very short integration times – sub-millisecond for ITDs and in the order of a few milliseconds for ILDs (Brown & Tollin, 2016). The operation of these neurons is essential for unlocking the full potential of binaural hearing: precise sound localization and spatial release unmasking beyond the head-shadow effect, called binaural contrast (Dieudonné & Francart, 2019). If prerequisites on the input to these neurons are not met, especially in individuals with electric stimulation and/or very asymmetric hearing, they may miss those benefits, even under aided conditions (Dieudonné & Francart, 2020), with respect to spatial release from masking). With respect to sound source localization, unnatural or asymmetric stimulation with broadband stimuli results in a root-mean-square (rms) localization error of 50 to 70° in bimodal and 30° in SSD-CI listeners, where 75° corresponds to chance performance (Angermeier et al., 2021; Dorman et al., 2016). The better SSD-CI performance is likely caused by the monaural localization abilities of the NH ear, because even SSD patients without an implant perform similarly (e.g., Agterberg et al., 2014). Another reason for the localization difficulties of bimodal CI users could be, that bimodal CI users have mainly low-frequency hearing in the acoustic ear, and therefore little access to ILD cues which dominate at high frequencies, while SSD-CI users take advantage of these cues (Dirks et al., 2019; Dorman et al., 2015). Bilateral CI users on average localize with similar accuracy as SSD-CI users (Dorman et al., 2016), but arguably for a different reason. They have less asymmetry but miss the benefit of one very good ear that can exploit spectral cues. The better 50% of bilateral CI users in (Dorman et al., 2016) have an rms error of 10–25° and this range can serve as ambitious goal for a bimodal CI user after perfect mismatch compensation. In contrast, normal-hearing listeners have an rms-error below 10° and a negligible localization bias of 1° (Ausili et al., 2019; Dorman et al., 2016). The remaining difference between best bilateral CI users and average NH listeners is not due to missing spectral cues but due to an inability to exploit fine-structure ITDs in the 500–1000 Hz region that provide the most salient localization information to NH listeners (Mills, 1958).

The degree to which asymmetrically impaired patients can still benefit from binaural processing in the brainstem arguably depends on the fitting of their devices. The three critical fitting dimensions are level, latency, and frequency (band allocation in electric hearing and frequency compression in hearing aids). A mismatch in any one of these dimensions can obliterate the benefits of binaural processing. To make matters even more complex, the interaural difference in each dimension is frequency-band specific. For level - which is arguably the most important fitting dimension - it is not even clear what a “matched level” means and what the fitting goal should be. Furthermore, an ideal fitting for the level dimension must be more than simply adjusting an amplitude-scaling factor. Rather, the output amplitude has to be a function of baseline level, and, due to different compression and adaptation effects in the devices and in the auditory system, the optimal level mapping even depends on the short-term input history (e.g., Spirrov et al., 2020).

Asymmetric hearing can disrupt binaural fusion. Normal-hearing and most symmetrically impaired listeners take binaural fusion for granted: A single sound source is perceived as a single object. Without fusion, there are two acoustic objects, one at each ear. Fusion is not binary; it can be partial and it can depend on the stimulus. The fundamental importance of fusion for binaural fitting is that it alters even how the experimenter has to ask the question and even the fitting goal. For example, in case of fusion, there is just a single percept and hence a single loudness. There is no left loudness and right loudness in this case, and no possibility for actual loudness balancing when both ears are stimulated simultaneously (Shub et al., 2008). Reversely, without fusion, centralization is not a meaningful task. Kan et al. (2013) showed that binaural fusion decreases with an increase in frequency mismatch for bilateral CI users. It also decreases with increasing latency mismatch, related to the echo threshold in the precedence effect (Litovsky et al., 1999). Therefore, optimizing binaural fusion is a central fitting goal and at the same time, the degree of binaural fusion influences how to fit – a catch 22. Despite this central role, binaural fusion is not discussed much in the context of binaural fitting, causing the present text to go beyond simple reviewing but rather to elaborate and sometimes speculate about implications of fusion in several sections.

Given this complexity, it is not surprising that until now, bimodal and SSD-CI users are mostly fitted as if their contralateral hearing would not exist. Usually, only a coarse, broadband loudness matching is performed. In the context of research studies, compensation of mismatch has been addressed in all dimensions (Bernstein et al., 2018; Francart et al., 2009; Reiss et al., 2015; Zirn et al., 2015), but a comprehensive, simultaneous compensation of all dimensions has not so far been reported. The most involved attempts to correct for several dimensions therefore stem from studies on ITD sensitivity in bimodal and SSD-CI users (e.g., Francart et al., 2018). The present work aims at finding a path towards comprehensive mismatch compensation. However, it is unclear whether this should necessarily be the goal of fitting for all patients. If an interfering sound is picked up by the better ear, it can have a negative impact on binaural speech comprehension, as sometimes seen in asymmetric bilateral CI users (Bernstein et al., 2020; Goupell et al., 2018) and a very different goal must be set. These - admittedly important - cases are not considered in the present work.

In the following, we first review the sources for interaural mismatches and elucidate their relation to brainstem processing and subsequent perceptual consequences. Next, we list mismatch measurement techniques, and describe what exactly they measure, as well as their limitations, interdependencies, and efficiency. While these two sections have primarily a review character, the next two sections aim at providing a perspective for future directions: There we assume that we know the mismatch and then elaborate on strategies that are expected to reduce the interaural mismatches, while considering their side effects. The last section is an attempt to give a clinical outlook: What tools are going to be required, and which measurement and fitting parameters are expected to provide the best return on time investment, for each patient group? Each section contains a subsection for each of the three dimensions: latency, frequency, and level. The focus is on post-lingually deaf adults with no other medical issues.

## Causes of Interaural Mismatches and Their Perceptual Relevance

When speaking of interaural differences, this commonly refers to acoustic differences in the sound fields at the left and right outer ear. In the present context, however, where the left and right inner ear are stimulated by different modalities, we refer to an interaural mismatch as any type of left-right bias introduced by a hearing device and by the asymmetric auditory pathway. For a sound that is emitted frontally, i.e., with no acoustic differences between the two outer ears, the bias can refer either to a different left-right auditory nerve (AN) response, or to a perceptual lateralization bias.

### Causes of Latency Mismatch

**Figure 2.**
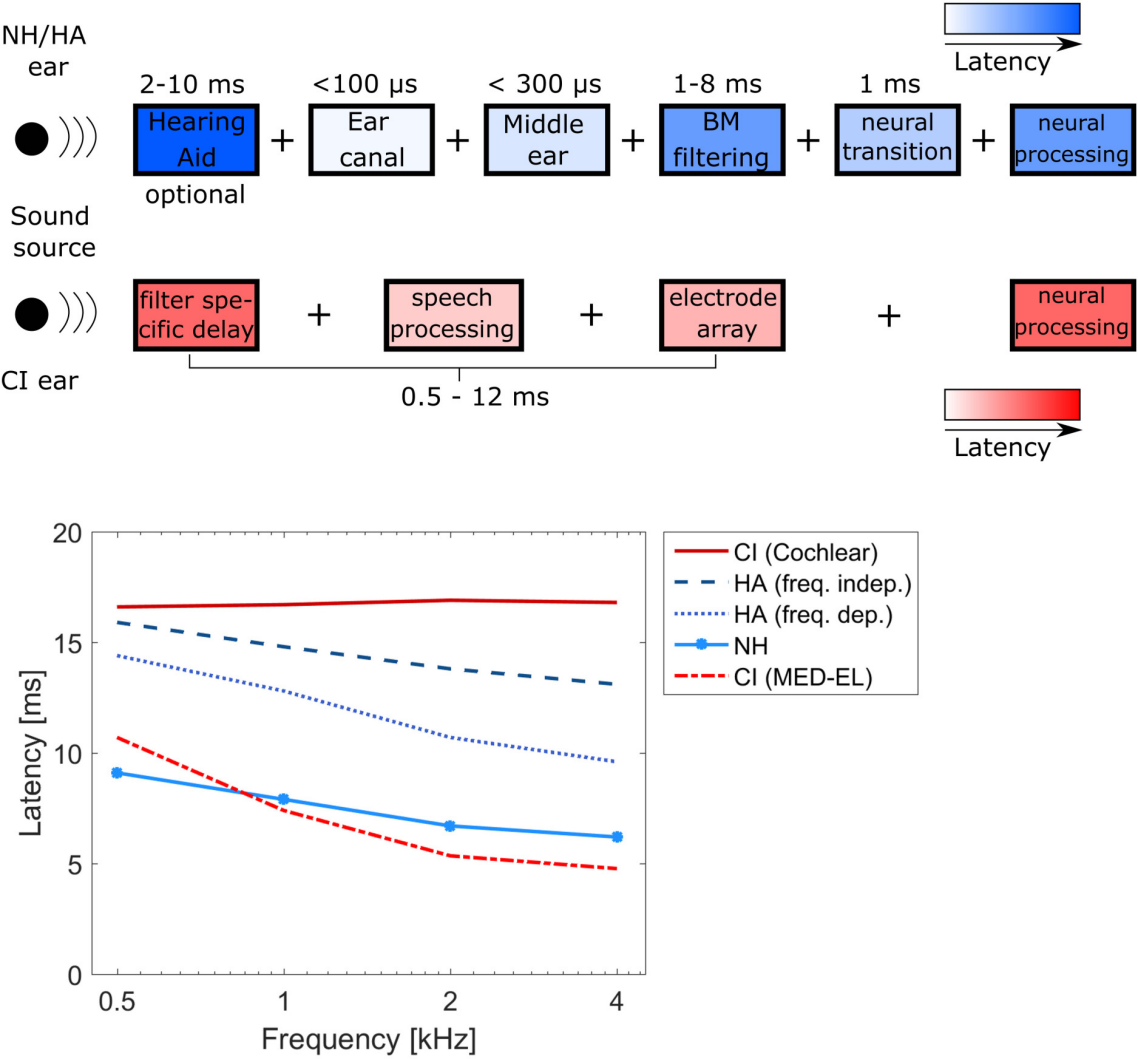
Upper panel: elements contributing to the peripheral latency and its mismatch between the acoustically and the electrically stimulated ear. Lower panel: Examples of wave V latencies (Normal-hearing (NH), CI (MED-EL), and HA (freq. indep.) data from Zirn et al. (2015); CI (Cochlear) and HA (freq. dep.) data from Engler et al. (2020)).

Sound processing along the auditory pathway requires a certain amount of time. In a normal-hearing listener, this processing latency is identical for the left and right ear. Assuming the same devices on both ears, the processing latencies for bilateral CI users should also be identical between ears, and they therefore will not suffer from latency mismatches. However, in SSD- and bimodal CI users, the early stage of the auditory pathway up to the inner ear is replaced by the electrical pathway of the CI system on one side. The most relevant peripheral contributions to the latency are HA processing and the inner ear on the acoustic side, and the CI processor on the electrical side (Figure 2).

With acoustic hearing, the sound wave arriving at the outer ear needs about 74 µs to travel through the ear canal and up to 250 µs to cross the middle ear (Gan et al., 2004). In relation to the total peripheral latency, both structures play a minor role. Much more important is the inner ear and the subsequent neural processing. The traveling wave along the basilar membrane causes a dispersion, i.e., short latencies of about 1 ms for high frequencies, but about 8 ms for the lowest frequencies (see, e.g., Ruggero & Temchin, 2007 for an review). The dispersion is particularly prominent at low frequencies. Due to the movements of the basilar membrane, the hair cells are stimulated and neurotransmitters are released to excite the auditory nerve fibers. This process takes approximately 1 ms and is independent of frequency (Temchin et al., 2005). Afterwards, the neural processing up to the auditory cortex requires several dozens of milliseconds, but this is not in the focus here, because from there, the pathways are the same for all stimulation modalities and thus latencies can be expected to be similar for a healthy auditory system (beyond the cochlea), despite minimal differences still being possible (Polonenko et al., 2015). Next to frequency, level has an influence on the acoustic latency as well. Both effects can be observed in measurements of the wave V in auditory brainstem responses (Neely et al., 1988). Between levels of 20 and 100 dB SPL and frequencies between 0.25 and 8 kHz, the latency of wave V decreases with increasing level as well as with frequency. The level dependence is assumed to occur due to summation effects of the individual neural responses after the non-linear processing of different levels through the basilar membrane (Ruggero et al., 2007).

When listening with a HA, a processing latency is added to that of the auditory pathway. Depending on HA brand and type, processing latencies vary between 2 and 10 ms. Some devices have a constant latency, while others are frequency dependent (Balling et al., 2020). Higher aided levels may slightly reduce the ear-processing latencies, and in the case of an open fitting, the direct path may need to be considered at frequencies with little amplification. Similarly, for the electrical ear, the CI device processing latency has to be considered. Depending on the type of filter bank employed, this may be fairly frequency independent in the case of Fourier transform-based filters (Tabibi et al., 2017), or it may approximate the traveling-wave dispersion of a healthy cochlea in the case of a time-domain-based filter, e.g., finite impulse-response (FIR) filters (Mahalakshmi & Reddy, 2010). Typical values are 12 ms for fast Fourier transform (FFT)-based (Engler et al., 2020; Wess et al., 2017), and 0.57 to 7 ms, from 4000 down to 500 Hz for FIR filters, respectively (Zirn et al., 2015).

**Figure 3.**
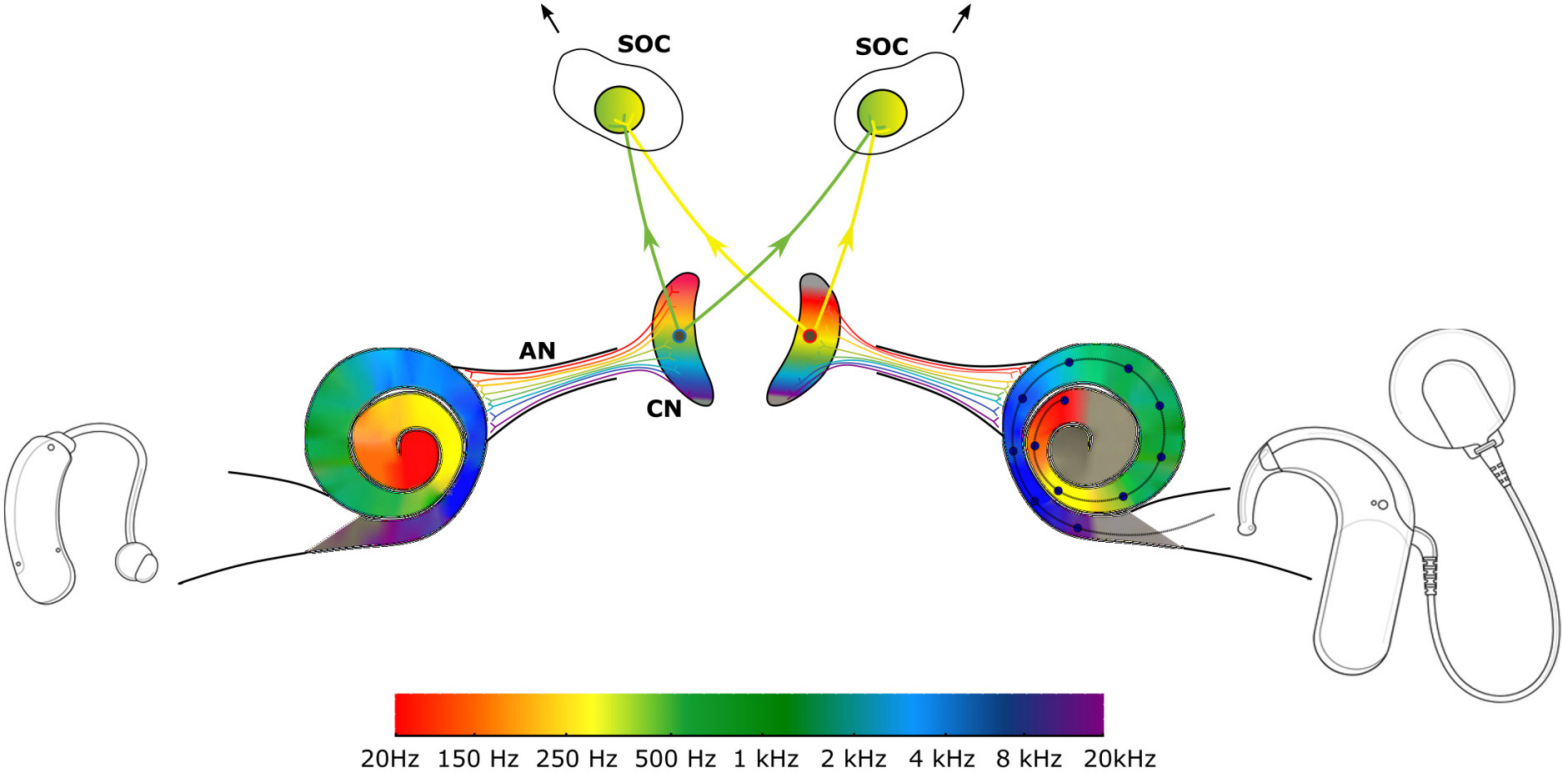
Frequency mismatch in bimodal ci. A binaural neuron in the SOC is innervated from identical cochlear positions and therefore from different frequency bands, as indicated by the different colors. AN: auditory nerve, CN: cochlear nucleus, SOC: superior olivary complex.

In the case of SSD-CI users, and provided there is a comparable neural processing latency, the FIR filter-based CIs cause a fairly low overall latency mismatch between acoustic and electrical hearing, as the FIR latency is comparable with the delay of the inner ear (Figure 3, lower panel). Consequently, bimodal CI users with a FIR filter-based CI usually have a large latency mismatch, approximately corresponding to their HA latency. Any latency mismatch is obviously an offset ITD.

Irrespective of any latency mismatch, bimodal and SSD-CI patients have comparatively poor ITD sensitivity. Even under controlled conditions, when interaural mismatches have been at least partially compensated for, median ITD detection thresholds are 438 µs for SSD-CI users when using optimal stimuli (Francart et al., 2018). This number is about four times higher than for average bilateral CI users (Laback et al., 2015), and 20 times higher than in untrained, young normal-hearing listeners (Thavam & Dietz, 2019). Given that even bilateral CI users barely exploit ITDs for sound localization under natural listening conditions (Seeber & Fastl, 2008), it can be expected that most bimodal CI and SSD-CI users would not be able to do so either, even if all interaural mismatches were compensated for.

That said, there are two other benefits of latency compensation. First, even if CI users may not be able to discriminate naturally occurring ITDs of up to 700 µs, a larger latency mismatch may lead to a constant lateralization bias towards one side of the lead (Williges et al., 2018). Secondly, more robust ILD sensitivity and binaural fusion are facilitated by having interaurally coherent input (Brown & Tollin, 2021). In the case of broadband stimuli, the temporal coherence is given by the filter bandwidth (Wiener Khinchin theorem). While narrow auditory filters provide a good coherence over several milliseconds, the wider analysis filters of CIs, and especially the effective channel bandwidths seen by the AN fibers, are several times broader (Frijns et al., 2001). For a bandwidth of 500 Hz, for example, not untypical for the middle region, interaural coherence vanishes within 2 ms of latency difference (Dietz & Ashida, 2021). It can be expected that larger latency differences lead to reduced ILD sensitivity and to a reduction in binaural fusion (Körtje et al., 2021). The limited fine structure ITD sensitivity and the slow envelope fluctuations in many natural stimuli such as speech, however, may cause a much longer envelope coherence length and thus latency to be less critical (e.g., Litovsky et al., 1999; Wess et al., 2017).

### Causes of Frequency Mismatch

The auditory pathway is organized tonotopically. Binaural neurons in the brainstem are innervated by tonotopically matched inputs (Batra & Yin, 2004; Joris et al., 1998; Smith & Delgutte, 2007), although small deviations are possible (Joris et al., 2006). In normal-hearing listeners, this is critical for ITD processing, because the temporal comparison of two inputs with different frequency content cannot result in meaningful fine structure ITDs, but only in fast-beating meaningless ITDs. Even for envelope-based cue processing, which is more relevant for CI users, e.g., in the lateral superior olive (Joris & Yin, 1995), an interaurally coherent input is required and tonotopically matched inputs are the only way to ensure this. Of course, speech and other common sounds have a substantial amount of co-modulation across a broader frequency range, so that even mismatched channels can provide exploitable cues.

In any case, it is a reasonable assumption that binaural hearing benefits fairly substantially from a tonotopic match of inputs. This is backed by a diverse set of studies: NH envelope ITD detection thresholds increase by a factor of 3 when the carrier frequencies differ by 10% between the ears (Nuetzel & Hafter, 1981). In bilaterally implanted cats, electrode contacts that are interaurally matched in cochlear position lead to the largest binaural interaction component (BIC) and to aligned stimulation patterns in the inferior colliculus (Smith & Delgutte, 2007). In human bilateral CI users, there is evidence that the ITD sensitivity is best with electrode contacts that also elicit the largest BIC (Hu & Dietz, 2015). This may have been expected, but when relating either of these two methods with the contacts that result in matched pitch, there can be an offset, and the correlation is weak (Hu & Dietz, 2015). The latter supports the assumption that place-pitch is plastic (Aronoff et al., 2019; Reiss et al., 2011), but especially in the post-lingually deaf, the inputs to binaural neurons are considered to be tonotopically hard-wired, i.e., not subject to plastic changes. Data from SSD-CI users also appears to agree with this assumption (Bernstein et al., 2018).

While SSD- and bimodal CI listeners benefit from the head-shadow effect, and their sound localization is improved compared to unilateral CI users, several other binaural benefits remain very limited, in part due to the frequency mismatch. Compromised binaural benefits include binaural fusion (Goupell et al., 2013), ILD sensitivity (Laback et al., 2004), binaural unmasking (Goupell et al., 2018; Sagi et al., 2021; Xu et al., 2020) and the separation of congruent speakers (Bernstein et al., 2016). Possibly a closer alignment between electrical and cochlear place frequency might improve these binaural benefits, and might even support a faster improvement of speech comprehension after implantation (Buss et al., 2018).

There are several possible causes for an interaural frequency mismatch. Arguably, the main reason in bimodal and SSD-CI listeners is that the standard frequency allocation is deliberately offset to the normal frequency-place transformation of the basilar membrane (Figure 3). The offset can be meaningful in bilaterally deaf patients by whom the implant's standard frequency range is optimized for speech perception, and starts below 200 Hz. Even for deeply inserted electrode arrays, the position of the most apical contact of an electrode array does not often correspond to such a low frequency. On average, the place mismatch between the allocated frequency of the given electrode contact and the Greenwood frequency is 4 to 5 mm (Landsberger et al., 2015), corresponding to approximately one octave, with large inter-individual differences. Other studies found average mismatch values around half an octave for different electrode lengths at the base with increasing mismatches of about 1 to 2 octaves towards the apex for shorter electrodes (Bernstein et al., 2021; Canfarotta et al., 2020). However, there is ongoing discussion about which acoustic place-frequency map the electrical stimulation should be compared to. The Greenwood function maps the place along the basilar membrane or along the spiral ganglion (Stakhovskaya et al., 2007) to the corresponding most sensitive frequency at threshold. As acoustic level increases, however, the center of the activation shifts towards the base. For bimodal patients with an outer hair-cell loss, the activation pattern may always have a basal bias. At intermediate sound levels, the shift can be as large as half an octave (Chatterjee & Zwislocki, 1997). Accordingly, Sagi and Svirsky (2021) proposed that a half-octave shift has to be considered for a more faithful place-frequency comparison. This half-octave shift leads to frequency allocations closer to the standard frequency-to-place allocations of the CI devices.

Simulated bilateral CI users showed reduced spatial release from masking for interaurally mismatched electrodes (Xu et al., 2020). In simulated SSD-CI users there is evidence, that a compensated frequency mismatch improves contralateral unmasking in noisy speech for initial mismatches larger than 3.6 mm along the cochlear place (Wess et al., 2017). Studies in bilateral CI users indicated that the binaural processing might be tolerant to mismatches up to about 3 mm (Kan et al., 2013; Poon et al., 2009), due to the large spread of excitation. This is a smaller range, than the average mismatch found in SSD and bimodal CI users (see above). Therefore, the mismatch in a majority of CI users is still a problematic issue. However, the studies investigating mismatch tolerance mostly used single electrode stimulation and little is known, how the tolerance is affected, when stimulating the whole electrode array. Additionally, things might be different for bimodal and SSD-CI users, as the acoustic side produces a less broad spread of excitation.

In addition to this main effect, several individual variations, such as deactivated electrodes, neural dead regions, and the morphology of the cochlea have to be considered as causes of individual frequency mismatches.

### Causes of Level Mismatch

**Figure 4.**
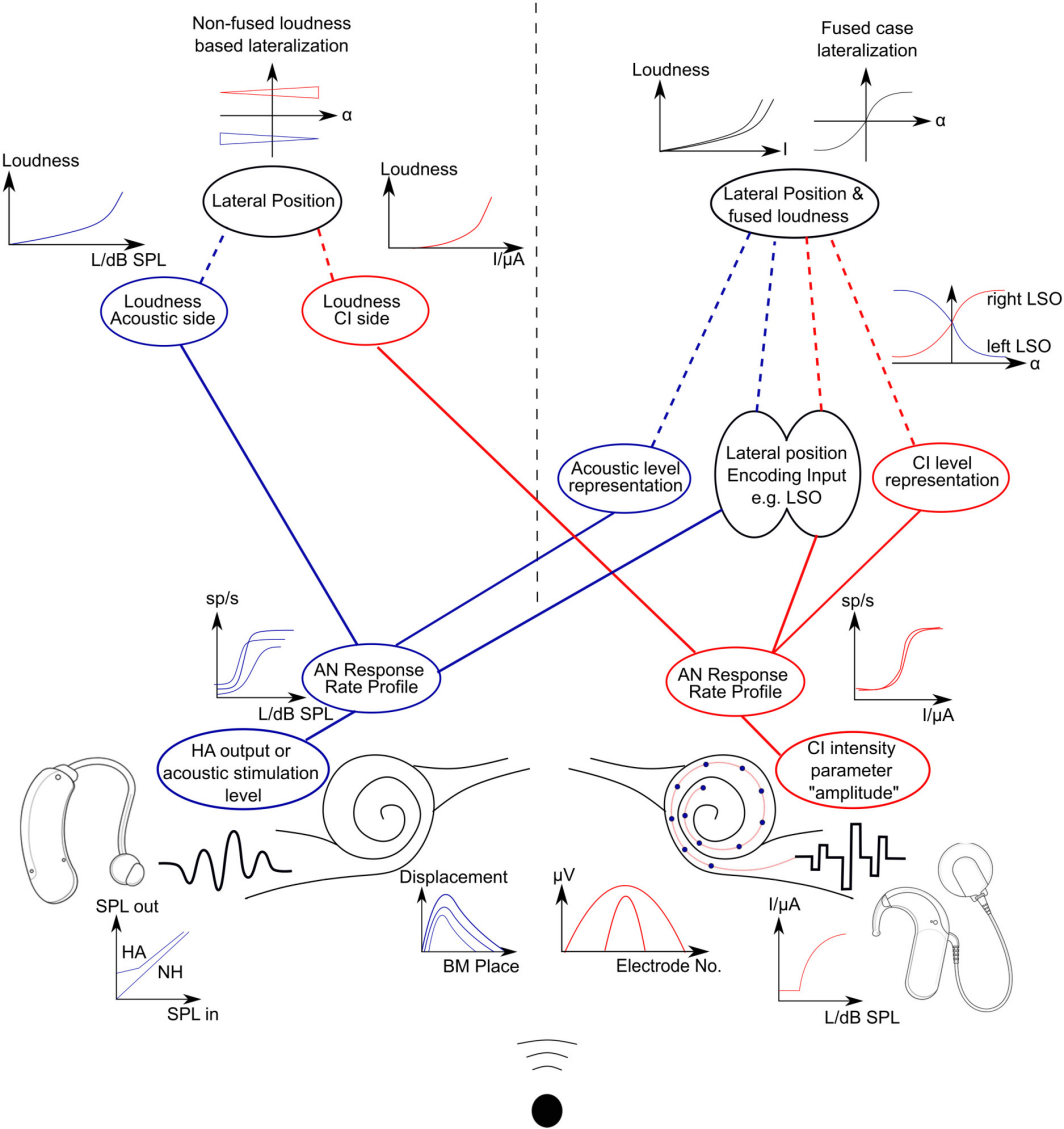
Transformation of acoustic sound level by the device and its encoding and decoding along the auditory pathway. The upper left branch illustrates decoding without binaural fusion, whereas the upper right branch illustrates decoding in case of binaural fusion. Each processing step can be understood as a complex transformation, usually with an imperfect correlation. BM: basilar membrane, CI: cochlear implant, HA: hearing aid, LSO: lateral superior olive, NH: normal hearing, α: azimuth of sound source.

As mentioned in the Introduction, it is already difficult to define a goal in fitting for level, and to define what “interaurally matched levels” actually refers to. Therefore, before being able to address causes of level mismatch, we have to clarify the coding of level in a bimodal and thus binaural context: At any earlier stage than the AN, i.e., at the devices’ outputs, a level difference is not defined, because of the different modalities. At the AN, sound level is encoded by neural response rates in both individual fibers and the ensemble of fibers. However, stimulation level is not necessarily directly related to the total response rate of all fibers, and also not necessarily to the average response rate per fiber. Similarly, the electrically evoked compound AN response (ECAP) is not a direct correlate of loudness (Kirby et al., 2012). Ultimately, when the left and right ear are stimulated together and the inputs fuse into a single auditory object, there is no longer a left or right loudness. Sequentially presented equally loud left and right stimuli, however, may result in a lateralization bias when presented simultaneously (Baumgärtel et al., 2017; Fitzgerald et al., 2015; Stakhovskaya & Goupell, 2017). For these two reasons, even an interaurally matched loudness may not be an appropriate level-fitting goal. In case of a binaurally fused percept, the fitting goal to optimizing binaural benefits should rather be that a frontal source is perceived centrally, i.e., not biased to the left or right (Figure 4). We do not write “is perceived frontally”, because CI users are not expected to have an externalized spatial perception (Best et al., 2020; van Hoesel et al., 2002). Furthermore, it has to be taken into account, that this fitting goal might compete with other goals, such as maximizing speech intelligibility.

Due to the asymmetric status in bimodal or SSD-CI users, the two ears can be expected to have a different number of AN fibers and/or a different tonotopic distribution of fibers. As such, a comparable stimulation leads to different compound responses, even if isolated fibers respond similarly. Secondly, acoustically stimulated AN fibers have very different sensitivities, resulting in dissimilar rate-level functions. Such a range of properties is not observed with electric stimulation (Huet et al., 2019; Miller et al., 2006), so that the distribution of response properties is probably always different across the modalities. Further, the electrical stimulation usually has a larger spread of excitation, compared to the rather narrow frequency bands of the acoustically stimulated basilar membrane.

Due to the hair-cell synapse, spike-rate adaptation on the acoustic side is stronger, as are adaptive gain-control mechanisms, especially via the efferent innervation of outer hair cells. This causes dynamically changing interaural response rate differences and differently steep level-growth functions resulting in interaural differences in loudness growth (McDermott et al., 2003). Both HAs and CI speech processors partly compensate for these problems by means of automatic gain control (AGC) algorithms and compressive mapping of acoustic to electrical level. However, this is a highly complex topic on its own and will be only coarsely treated in the following. The normal- or unaided hearing brain can be informed about peripheral gain-control settings, i.e., when forming percepts that depend on level or ILDs, it can consider which gain-regulating reflex is active at any moment. A hearing device, on the contrary, only provides the modified input but cannot communicate its settings. The brain therefore has to take the inputs at face value and may misinterpret stimulation levels, even if the AN response rates are matched.

To date, HAs often operate with frequency-channel-specific AGCs (Hohmann, 2008), whereas CI speech processors more often operate with broadband AGCs (Vaerenberg et al., 2014). In addition, the independent configuration programs in both the CI and HA devices mean different amplification modes, changing in an adaptive way, usually without any communication between the sides. In bilateral CIs and binaural HAs, synchronized AGCs have been shown to reduce this problem (Pastore et al., 2021; Sockalingam et al., 2009), but this technology will not alleviate all of the above-described differences in bimodal CI users (Spirrov et al., 2020).

Yet, another reason for response rate differences comes from general stimulation or excitation limitations. The impaired acoustically stimulated cochlea may not be sufficiently sensitive at the basal end, whereas electric stimulation may be limited near the apex or to avoid unintentional electrical excitation of the facial nerve (Seyyedi et al., 2013; Smullen et al., 2005). As a consequence, level-related percepts will have a different spectral profile in each ear, ultimately resulting in a frequency-dependent interaural level and loudness difference (Buss et al., 2018).

The long list above focused on differences manifested as different AN response rates. As noted at the beginning of this subsection, perception may not necessarily reflect these rates. Especially when the left and right stimulation fuse into a single object, we expect a very different situation than without fusion. Without fusion, we perceive two separate images and an interaural level mismatch may cause a loudness imbalance (Figure 4, upper left). In the more desired case of binaural fusion, we expect that subcortical binaural neurons are able to compare and integrate the two sides, but in a very different way to when we compare left and right loudness in the absence of fusion. These neurons can be expected to compare within frequency bands (Batra & Yin, 2004; Joris et al., 1998; Smith & Delgutte, 2007) and on very short time scales (Joris, 2019). As such, a balanced level within the overlapping frequency region is critical.

## Mismatch Measurement Techniques: Efficiency, Limitations, and What is Actually Measured

To be able to compensate for the interaural mismatches and to facilitate an improvement of binaural benefits (see section “mismatch compensation and side effects”), one has to first find the proper measurement tools to determine the amount of the respective mismatch. In this section, we summarize the current possibilities to do so and point out their advantages and their limitations.

### Latency Mismatch Measurements

To determine the interaural mismatch in latency between the acoustic and the electric ear, the processing time of each side has to be known. This can be achieved by determining the processing time of the auditory pathway including the hearing device, or by determining the processing time of each component separately, i.e., the hearing device latency and that of the auditory pathway in separate measurements. The prime measurement method for both possibilities is the measurement of the wave V latency via (e)ABRs. As long as the auditory pathway is responding to the stimulus, wave V is a robust peak that can be identified in the brainstem response even with electric stimulation, and therefore allows for a reliable prediction of the processing latency along the auditory pathway (Firszt et al., 2002; Neely et al., 1988).

The combined device and patient latencies of the acoustic-hearing side, can be measured in a free-field environment, e.g., using loudspeakers, or by HA via audio cable (Zirn et al., 2015). The two suggested stimulus types, narrow-band chirps and tone bursts, have been found to lead to wave V latency differences of several milliseconds (Cobb & Stuart, 2016; Rodrigues et al., 2013). However, this difference is only due to the different definitions of stimulus onset for the two types (Cobb & Stuart, 2016), emphasizing the importance of all temporal definitions in this context. The advantage of assessing the acoustic pathway, including the HA latency, is that only a single measurement is required. However, with increasing hearing loss, the identification of wave V becomes more difficult for ABR measurements that include an unknown HA latency (Dawes et al., 2013). Therefore, it might be easier to determine the HA latency separately from the ABR measurement with a hearing-aid test box or equivalently performing tools (Angermeier et al., 2020). Thereby, the HA latency is measured by the input/output difference of a particular point on the envelope's rising flank or can be determined by the lag of the maximum of the cross-correlation function. With a separate measurement, the largest source of variance, the device latency, can be measured accurately without a human listener, while the auditory pathway latency is established using direct stimulation via headphone.

For the electrically stimulated side the same question arises; whether to measure the whole system, or device- and neural latencies separately. In the case of a combined measurement, CI pulses can be emitted during the moment that the brain response is occurring. To avoid these pulse artefacts that are much larger than the brain response, a stimulus design that knows how the sound-processing strategy behaves is needed. The more direct way forward is to stimulate the CI electrodes directly and measure the neural latency in isolation. Pulse artefacts will still be present, but precede the response. Even if the artifact leaks into the response time window, it is fully deterministic in the case of direct stimulation and methods are available to subtract it (e.g., Hu et al., 2015).

The CI device latency may not be known, but if latency mismatch compensation is going to be part of the bimodal fitting process, it can be expected to have been taken into account by the manufacturer-specific fitting software. In the meantime, CI device latency can also be determined by direct electric measurements at the CI electrodes (Zirn et al., 2015). The pulsatile output provides an even clearer definition of the response moment than the HA output. However, as the response-time definitions cannot be identical for the two modalities, this imposes a source of error for the isolated latency measurement approach.

Overall, wave V-based latency measurements are possible and informative. For the most important frequency-specific latencies, however, errors larger than 1 ms can often not be avoided. Additionally, clinics might not have access to (e)ABR settings to reliably measure wave V-based latencies. Taking these two points into account, average (e)ABR latencies, or estimations from an individualized simulation (Verhulst et al., 2016) offer a viable alternative, without any actual ABR measurements. As in the separate measurements case, the individual device latencies would need to be added.

### Frequency Mismatch Measurements

Different approaches have been tried to determine the specific interaural tonotopic mismatch. The different measurement techniques are psychoacoustic, image-based, or based on the BIC derived from auditory brainstem responses. In this section, we discuss the benefits and the limitations of these methods.

The arguably fastest measurement technique is imaging. With the help of x-ray or computed-tomography (CT) images, the electrode position within the cochlea can be measured by estimating the insertion angles of the electrode contacts. Due to the tonotopic order of the cochlea, the insertion angle can be assigned to a specific center frequency using the Greenwood (1990) frequency-position function (Boëx et al., 2006; Cohen et al., 1996; Landsberger et al., 2015). Applying the Greenwood equation, the frequency range along the organ of Corti, as well as a spiral-ganglion related frequency position can be represented (Stakhovskaya et al., 2007), and potentially a correction term for the level-dependent acoustic activation shift has to be considered (see “causes of frequency mismatch”). We do not expect that these corrections need to be measured in each individual, so they are not considered in this section. When using x-ray images, additional aspects, such as the image quality have to be considered. The quality of the x-ray images differs, depending on correct exposition and exposure parameters, leading to artifacts or insufficient contrast of the image (Kirberger, 1999). This might hamper the correct determination of the electrode contacts’ insertion angle and its corresponding center frequency within the cochlea. CT images may not be available in all clinical protocols, and their acquisition causes a much larger radiation dose than the recording of x-ray images, which has to be taken into account when planning to record CT images solely for the purpose of estimating the frequency mismatch. On the upside, it generates high-quality 3D images, which improve the assessment of the insertion angle and allows automatic estimation of the electrode position (Bennink et al., 2017; Canfarotta et al., 2019; Mertens et al., 2020). With good quality images, the mean absolute error of x-rays is 12.6° (10.6% relative frequency error), and for CT scans it is 9.7° (8.1% relative frequency error), both compared to histological data (Gallant et al., 2019). This is quite precise, compared to the deviations discussed in “causes of frequency mismatch”, with CT scans being the most precise measurement available. As either x-ray or CT images are mostly part of clinical protocols for postoperative monitoring of the electrode position, no additional measurement of the patient would be necessary. An image-based frequency allocation, however, only accounts for the place of electric stimulation, not for the tonotopic place of neural activation. A local degeneration of the spiral ganglion will cause a deviation that this method cannot capture. Furthermore, neural morphology and orientation along the electric field gradient causes a deviation between electric field strength and neural activation away from the dendrite (Bai et al., 2020). Both effects cannot be captured using imaging. However, comparing the different measurement techniques with each other, good agreement between CT-images and ITD sensitivity (see below) has been reported (Bernstein et al., 2021), suggesting a good reliability of estimating the frequency mismatch using imaging.

Even without the availability of an image, the position of electrode contacts and its corresponding frequency can be estimated. Therefore, the surgical information about the insertion depth and technical information about electrode length and spacing between electrode contacts can be used to calculate the position within the cochlea (Dirks et al., 2021). If available, this might be combined with pre-operative CT scans to determine the individual cochlear duct length. Otherwise, the amount of influence of the cochlear length on the insertion angle is unknown. Another factor that cannot captured without a post-operative scan and influences the insertion angle is the lateral wall position of electrode array. Angle estimation based on surgical information is not as precise as using imaging and there is a possibility that the electrode array slips right after surgery. Furthermore, the same disadvantages (other than radiation) as for imaging exist.

The most commonly employed behavioral method to measure tonotopic mismatch is pitch matching (Laback et al., 2015). The acoustic frequency is varied to elicit a pitch equal to that elicited by the stimulated electrode contact. The underlying assumption is that a certain pitch corresponds to a fixed tonotopic place. Compared to measuring ITD sensitivity, pitch matching is fast and easy. However, the test has several disadvantages and has to be designed very carefully to avoid non-sensory biases. First, the selected frequency range can influence the result and lead to matched frequencies that differ by more than a 2/3 octave (Carlyon et al., 2010). Second, the starting frequency of an adaptive measurement can influence the pitch-matching result (Carlyon et al., 2010; Schatzer et al., 2014), and third there is a statistical effect where the mean pitch match shifts away from the edge of the response range (Jensen et al., 2021). Further, the choice of test method has an influence on the result (Jensen et al., 2021) and pitch depends on the acoustic and the electric stimulus types and its properties, rendering any match stimulus-specific (Adel et al., 2019; Lazard et al., 2012). Last, a level dependence can be expected, and likely has a fundamental but complex origin (Sagi & Svirsky, 2021). Apart from these important multiple possible procedural biases, there is an additional critical limitation of pitch matching: The brain appears to adapt the pitch percept for each electrode to the respected programmed frequency bands already within the first months after the first fitting (Reiss et al., 2015). Comparing different measurement techniques to determine the interaural frequency mismatch, in experienced bilateral as well as SSD-CI patients, also hints at such plasticity effects in electric place pitch (Bernstein et al., 2021; Hu & Dietz, 2015; Staisloff & Aronoff, 2021). In this case, the pitch matching might be an appropriate approach only for newly implanted CI patients, but potentially misleading for experienced users. Even for newly implanted patients, previous impaired acoustic hearing could have biased place pitch. Overall pitch matching does not appear to be suitable to estimate the mismatch for the purpose of improving binaural hearing.

Measuring ITD sensitivity while varying the place of stimulation in one ear builds on the finding that a tonotopically matching stimulation results in maximum ITD sensitivity (Nuetzel & Hafter, 1976). In bilateral CI users, this technique has been shown to produce the expected results (Hu & Dietz, 2015; Poon et al., 2009; Staisloff & Aronoff, 2021). Together with ILD sensitivity, it is arguably the most direct measure of interaural frequency mismatch, as long as the goal is to maximize this binaural sensitivity. As a downside, the method is very time consuming, and even with bilateral CIs only about 90% of the tested subjects are ITD sensitive (Laback et al., 2015). This fraction is possibly even smaller in bimodal or SSD-CI users (Bernstein et al., 2018; Francart et al., 2009).

Another difficulty that arises with the transition from bilateral to bimodal is that latencies may no longer be matched, causing an extreme bias to any static ITD task. This problem can be reduced by first matching the latencies (see section “latency mismatch compensation” and “latency fitting”) or by using non-singular ITD values, such as a large range of different, fixed ITDs (Bernstein et al., 2018), or dynamically varying ITDs (Dirks et al., 2020). The important positive aspect of ITD sensitivity testing is that it appears not to be affected by plasticity as pitch (Bernstein et al., 2021; Hu & Dietz, 2015; Staisloff & Aronoff, 2021), because it arises from presumably hard-wired binaural interaction at the level of the brainstem. Overall, as a binaural task, this method appears to be the most direct towards the question how to measure a mismatch to improve binaural processing (in contrast to CT imaging being the most precise), but is time consuming and presumably challenging for many - and impossible for some - bimodal subjects.

Last, it is possible to determine the tonotopic alignment using the BIC derived from auditory brainstem responses (Hu & Dietz, 2015; Smith & Delgutte, 2007). Most commonly, a wave V-related BIC is extracted from three ABRs: the difference between the binaural ABR and the sum of the two monaural ABRs (Levine, 1981). It arises primarily from excitatory-inhibitory interaction at the level of the lateral superior olive (Laumen et al., 2016) and is thus an ideal objective measure for the strength of binaural processing in the brainstem. The BIC does not require experience or training for the listener. However, difficulties arise from the technically challenging, electrically evoked ABR recordings, due to the large electric artifact and the small neural signal (Hu et al., 2015). In addition to conventional ABR, the BIC is a difference potential where absolute errors are larger than in a regular ABR, whereas its amplitude is usually less than 50% of the ABR wave V amplitude. For using it as a tool to contrast the BIC of neighboring electrodes, very careful and long recordings have to be conducted, and so far, only one study was able to quantify interaural mismatch with this method in bilaterally implanted humans (Hu & Dietz, 2015). Even in normal-hearing listeners, it remains a challenging task (Sammeth et al., 2020). With bimodal and SSD-CI users, the task is presumably even more challenging. As for ITD sensitivity, the interaural latency and level differences might have to be corrected first to correctly align the acoustic and electric ABR responses. However, the possibility of asymmetries for wave III-V interpeak latencies between acoustic and electric ABRs in bimodal CI users makes it difficult to get a BIC in the first place (Polonenko et al., 2015), let alone to quantify amplitude differences for neighboring electrodes.

### Level Mismatch Measurements

Various behavioral and evoked-response based measurements have been suggested to derive the interaural level mismatch in CI users (Balkenhol et al., 2020). Most commonly “loudness balancing” is the reported fitting goal and as such also the mismatch measurement technique (van Eeckhoutte et al., 2018; Veugen et al., 2016a). However, just because this term is used does not mean that loudness balancing was actually performed (see section “causes of level mismatch”). Sometimes, the subjective task that is actually conducted and called “loudness balancing” is arguably better described as a centralization task: Patients are stimulated simultaneously in both ears and have to report their perception, such that the presence of a lateral bias can be detected (Stakhovskaya & Goupell, 2017). In the absence of binaural fusion, this can be described as loudness balancing, and patients instead report whether the two signals at the left and right ears are perceived as being equally loud. This loudness balancing can be performed either sequentially or simultaneously. With binaural fusion, however, an interaurally balanced loudness that is defined using sequential stimulation often does not produce a centralized sound perception and is, instead, biased towards one ear (Baumgärtel et al., 2017; Florentine, 1976).

In clinical fitting, the loudness balancing or centralization task is normally done while listening with both ears to a relevant broadband signal such as speech or speech-shaped noise, and typically only for one signal level. For psychoacoustic research or for frequency-specific compensation, it is also possible to perform the task with single electrodes and a more narrowband acoustic signal. However, it might be necessary to measure and compensate for the interaural frequency mismatch first, as frequency ranges of the electrode-to-frequency allocation might shift during the compensation process (see section “frequency mismatch measurements” and “frequency mismatch compensation”). To match levels across the dynamic range, a direct measurement becomes very time consuming. Adaptive measurement techniques (Brand & Hohmann, 2002), or model-supported measurements of loudness perception are possible, and vastly increase efficiency (Francart & McDermott, 2012). The latter uses tone complexes of different bandwidths to avoid separate measurements for each frequency channel.

A very different approach is to use evoked, response-related measurements, allowing level estimates and balancing at specific stages of the auditory pathway, for example in the brainstem and midbrain by means of auditory brainstem response (ABR) amplitudes (van Eeckhoutte et al., 2018) or late auditory evoked potentials (LAEP), which are more strongly correlated with loudness (Hoppe et al., 2001). Similar to the loudness-scaling procedure, obtaining level-mismatch data via evoked-response-related measures is highly time consuming. Finally, the adequacy in loudness perception when evoked-response-based measurements are used remains uncertain. Kirby et al. (2012) demonstrated that stimuli that evoke equally large amplitudes in the left and the right ear of a bilateral CI user are not necessarily perceived as equally loud.

## Mismatch Compensation and Side Effects

The starting assumption is that the patient has a mismatch in latency, frequency, and level and that these mismatches even vary with many stimulus and device parameters; most of all they differ for each frequency band. When trying to compensate these three mismatched dimensions, one needs to be aware of interdependencies, e.g., a change in HA gain also changes the wave V latency, due to the level dependency of ABR latencies (Werner et al., 1994). Another problem is that if one dimension has a large mismatch, this mismatch may severely impair sensitivity to a change in the other dimensions. For example, in normal-hearing listeners with vocoded stimuli, a large latency difference strongly reduced their sensitivity to a frequency mismatch, i.e., compensating one mismatch, without considering the remaining dimensions at the same time is not sufficient to reach the optimal outcome (Wess et al., 2017). This imposes a large additional challenge to a task that is already difficult for each dimension in isolation. However, it also underlines the importance of compensating all three dimensions. This section describes the pros and cons of compensation strategies for each of the three dimensions. It also elaborates on the side effects or inter-dependencies, because they should influence the order and structure of a clinical fitting protocol (section “clinical outlook”).

### Latency Mismatch Compensation

As discussed in section “latency mismatch measurements”, the goal is to match latencies between sound arrival and a certain neural biomarker, such as the ABR wave V. Latencies can only be matched by increasing the device latency on the side with the shorter latency. In practice, most devices do not allow for a latency adjustment, and if they do, it is a frequency independent delay. In this subsection, we describe how the latency would have to be adjusted in the optimal case and ignore the present device limitations. The type of adjustment depends primarily on the CI processing type (FFT- or FIR-based filter bank) and the acoustic processing (no HA, frequency dependent HA latency, frequency independent HA latency). Compensation requirements resulting from the examples of latencies shown in Figure 3 (lower panel) are summarized in Table 1.

**Table 1.** Overview of Latency Compensation Possibilities for Different Combinations of Unaided and Aided Ears for SSD- and Bimodal CI Listeners.

	FIR CI	FFT CI
**unaided**	already almost equal / freq. specific delay CI	not possible
**freq dep. HA**	freq. specific delay CI	freq. specific delay CI/HA
**freq ind. HA**	constant delay CI	freq. specific delay CI/HA

In the case of SSD-CI users with FIR-based processing, the interaural latency mismatch is relatively small and only at high frequencies does the CI side have a slightly shorter latency (Zirn et al., 2015). Thus, the ideal compensation would be a CI delay at high frequencies only. However, even a constant delay of 1 ms improves sound localization and leads to a decreased rms angular error of only 10° (Seebacher et al., 2019). In contrast, the processing time in FFT-based CIs leads to a larger latency relative to acoustic hearing (Wess et al., 2017) and therefore cannot be compensated for.

In bimodal CI users, the HA processing latency is an additional component to be considered for latency mismatch compensation. The easiest cases are bimodal CI users with FIR-based processing on the CI side and frequency independent processing in the HA. As for SSD-CI users, despite the mismatch in latency, the electric as well as the acoustic side have a similar frequency-response curve that can be compensated for with an additional constant delay on the CI side. For some manufacturers, a latency compensation using a constant delay is already included into their fitting software, e.g., MED-EL Maestro 9. If bimodal CI users would be provided with a HA with a frequency dependent latency (in addition to the already frequency dependent inner ear latency), a frequency dependent delay on the CI side is ideal.

In bimodal CI users with an FFT-based CI, the device latency is frequency independent but relatively large. Depending on the HA latency, it is possible that the latency on the acoustic ear is shorter than on the CI ear. In that case, a frequency-dependent compensation has to be performed at the HA, as the latency difference can be expected to increase with increasing frequency. Another complication for bimodal CI users might be patients with an open HA fitting. In addition to the processed signal, the direct sound path may play a role, especially at low frequencies (Bramsløw, 2010) and with mild to moderate hearing losses. In bimodal CI users, this leads to a delay compensation that is not only frequency dependent but also dependent on HA gain: With less gain, the direct sound path dominates and the HA pathway plays a minor role. A compensation of HA latency might not be necessary or even have a negative impact at low frequencies. A direct sound compensation offered by some HA devices can further complicate the situation. Although less interdependence between latency and frequency compensation is expected, an adjustment of frequency specific delays at the CI might be necessary after frequency compensation, as the frequency ranges of electrodes might change (see section “causes of frequency mismatch” and “frequency mismatch compensation”).

In bimodal CI listeners, an acute compensation of the latency mismatch by adding a constant delay to the CI side was performed by Zirn et al. (2019) and Angermeier et al. (2021). After one hour of acclimatization to the added delay, the subjects showed a significantly decreased rms-error, resulting in an improved sound localization accuracy in the test situation of more than 11% and on average a reduced lateralization bias by 15° compared to no latency correction. Both studies are showing the importance of latency mismatch compensation for sound localization. With the CI side and the unaided acoustic side producing similar latencies, Zirn et al. (2019) solely compensated for the HA processing latency. Angermeier et al. (2021) where able to show an even better outcome using a delay compensation of HA processing latency plus an additional 1 ms for the difference between MED-EL CI and NH latency (Seebacher et al., 2019).

### Frequency Mismatch Compensation

In SSD-CI users, a compensation of the interaural frequency mismatch is not possible at the acoustic-hearing ear. Also in most mild- to moderately hearing-impaired patients it can be assumed that there is no frequency compression employed in the HA. Furthermore, in cases where the acoustic ear is the better ear, it would not be prudent to possibly distort the speech signal by doing compensations at the acoustic ear. Therefore, the compensation has to be implemented at the CI side. Normally, the frequency band delivered over the CI electrodes differs deliberately from the respective Greenwood frequency, to provide the complete frequency range (e.g. 150–8000 Hz) required for optimal speech intelligibility (Landsberger et al., 2015). However, it has been argued that this does not need to be the case for SSD-CI users and for good bimodal CI users (Sheffield et al., 2020). If these SSD-CI and bimodal CI users would be re-programmed to obtain tonotopically matched stimulation across the ears, they might lose low-frequency information on the CI side, depending on the insertion depth of the electrode array. However, in contrast to their purely electric-hearing peers, they obtain this low-frequency information from their acoustic hearing ear. With head shadow being small at those frequencies, they can access the low-frequency information independent of its direction of arrival. When the CI side is the poorer performing ear, discarding low frequencies up to 1000 Hz on the CI has been shown not to compromise speech intelligibility (Sheffield et al., 2020), emphasizing the value of considering the two ears as one hearing system, rather than treating the ears separately. Another approach was presented by Lambriks et al. (2020). Instead of discarding low frequencies, they use the possibility of the use of phantom electrodes in the CIs of Advanced Bionics, creating a virtual channel below the most apical electrode by simultaneous stimulation of the most apical electrode and a nearby electrode with opposite polarity. While the center frequencies of the electrode array are programmed to compensate the interaural frequency mismatch on an image-based approach, a phantom electrode is used to represent the discarded low frequencies in the CI ear.

Also the usability of evolutionary algorithms to optimize the frequency fitting is currently investigated and shows promising results concerning speech outcome and sound quality, whereas specific binaural benefits are yet unknown (Saadoun et al., 2022).

There are hints that bimodal CI users might be tolerant to small frequency mismatches and that for deep inserted electrodes, a compensation of the frequency dimension alone might not have a large impact on binaural benefits. With an average frequency mismatch of 0.15 octaves, Dirks et al. (2021) were not able to find significant changes in spatial localization or speech perception in different noise conditions in SSD-CI users after frequency mismatch compensation. However, an additional compensation of the remaining mismatches (e.g., latency) was not performed but might be necessary due to interdependencies between the different interaural mismatches.

Compensating the mismatch by shifting the frequency-to-electrode allocation, it is important to address deactivated electrodes or neural dead regions as well. It is well known that the existence of cochlear dead regions can constrain the benefit of combining acoustic with electric stimulation (Zhang et al., 2014). In these cases, two different strategies for mapping frequencies in cochlear dead regions are known. From a pure tonotopic matching perspective, the respective frequency region has to be discarded (dropped frequency mapping). Of course, this may severely impact speech intelligibility, and a compromise may be to reallocate the frequency band to the neighboring active electrodes (redistributed frequency mapping). There is no consensus regarding the impact of either strategy on speech perception. Some studies reported no significant difference in speech recognition between the two frequency remapping strategies (Shannon et al., 2002), others found that after several hours of training, speech identification could be considerably improved in the redistributed conditions (Smith & Faulkner, 2006). The disaccord might be related to the inaccuracy in finding the cochlear dead regions during the fitting session. Won et al. (2015) examined the influence of different remapping conditions on the spectral and temporal perception in CI users when different sizes and patterns of dead regions are present. The study did not reveal any difference in CNC word recognition. However, the spectral and temporal modulation-detection performance varied considerably between the strategies, suggesting that a trade-off between the spectral and temporal-envelope sensitivities might be beneficial. Further studies are required to assess the consequences of remapping in bimodal or SSD-CI patients.

If the latency of the filter bank of the CI speech processor is frequency dependent, an inter-dependence of latency matching and tonotopic matching has to be expected. This has already been observed in one bilateral CI user, where it was assumed that a tonotopic mismatch induced an otherwise absent latency mismatch (Williges et al., 2018).

### Level Mismatch Compensation

When conceiving a bimodal or any type of bilateral fitting, arguably the first thought is on adjusting the stimulation level. The most common fitting-goal description in this respect is “loudness balancing” (Ching et al., 2004; Francart & McDermott, 2012; Keilmann et al., 2009; Veugen et al., 2016a; Vroegop et al., 2019). Loudness balancing is arguably the most plausible goal in the absence of binaural fusion (see section “level mismatch measurements”). In the desirable case of binaural fusion, there is no isolated left or right loudness, and the fitting goal is rather a centralized perception. Note that a left and a right stimulus that are perceived as equally loud in isolation, do not necessarily result in a centralized percept (Florentine, 1976); see also section “causes of level mismatch” and “level mismatch measurements”). Irrespective of whether the goal is loudness balancing or centralization, achieving either for stimuli with differing spectra and at unlike overall levels is extremely complex, if not impossible, and partly ill-defined. This leads to the fact that a scientific and practical accord on how to achieve a compensation of the level mismatch has not yet been met.

On the other hand, reducing an interaural level mismatch may not be a desired goal in the first place. Especially in the absence of binaural fusion, there is apparent value in optimizing each device by itself in an attempt to maximize speech intelligibility (English et al., 2016). In cases of peculiar binaural loudness summation, a comfortable overall loudness has to be monitored and considered in any level fitting (Oetting et al., 2016). This is an even more critical concern in SSD-CI users, where a two-sided level reduction is not possible, as no control over the acoustic ear is given. Therefore, adjusting the CI to reach an interaurally balanced level may lead to a potentially uncomfortably loud percept. A third optimization strategy is to adjust level settings such that the two modalities complement each other in terms of frequency content, i.e. balancing the overall loudness across frequency bands rather than interaurally (e.g., Keilmann et al., 2009). This may be a prudent approach in bimodal patients with mostly low-frequency acoustic hearing, where a matched level between electric and an impaired acoustic hearing may not be possible for the very low and the very high frequency ranges. Especially in bimodal listeners that suffer from severe- to profound hearing loss at high frequencies, the CI dominates in the high frequencies, while the lowest frequencies are not transmitted. This will lead to a moving perception if a sound source changes in level or frequency composition. Without wanting to give the impression that these approaches are by any means inferior, the focus of this section is on interaural mismatch compensation. An overview of different steps for compensating the level mismatch is listed in Table 2.

**Table 2.** Overview of Adjustment Possibilities to Achieve Level Mismatch Compensation.

Findings	Adjustment possibilities
Fusion	Centralization
No Fusion	Loudness Balancing
Loudness Growth Mismatch	Adjusting compression ratios
Difference in Dynamic Range - no to moderate hearing loss 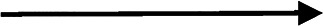 - severe hearing loss 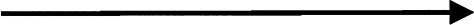	Adjust AGC parameters - gain control steps - time constant/knee point
Spectral dependence - no to moderate hearing loss 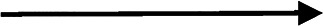 - severe hearing loss 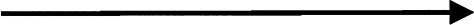	Narrow band signals - Centralization/Loudness Balancing - Balancing overall level across frequency

The two most common practices are left- and right-loudness balancing (e.g., Stakhovskaya & Goupell, 2017) and centralization (e.g., Litovsky et al., 2012). Both strategies are usually performed using a broadband signal (e.g., speech or speech-shaped stimulus) at intermediate levels, e.g., 70 dB SPL (Magalhães et al., 2021). A commonly used recommendation is to adjust the overall gain on the HA (Ching et al., 2004), but adjustments on the CI may be performed if the acoustic ear provides the best speech intelligibility and one does not want to compromise the corresponding HA settings.

In cases where the patient has a binaurally fused percept, fitting towards a centralized perception should be favored (see section “level mismatch measurements”). If the degree of binaural fusion is unclear, a centralization setup is still possible and arguably ideal, because even CI users with partial fusion or without fusion will be able, with the alternative instruction to match loudness instead of centering the sound image, to find the level at which both percepts are equally dominant or equally loud.

Due to different loudness growth between electric and acoustic hearing, the relative levels necessary to achieve a balance dependents greatly on the absolute level (Goupell et al., 2013), and can be expected to also depend on the spectrum of the stimulus. The absolute level dependence can be compensated for by adjusting compression ratios, and the spectral dependence by using narrow-band signals for frequency-specific compensation (Francart & McDermott, 2012). Avoiding mismatches in level compression seems to be crucial for the binaural benefits in congruent talker situations (Wess & Bernstein, 2019).

Additionally, dynamic aspects of the processing in the devices and in the auditory system may disrupt the ILD cues. Matching the parameters of the AGC, including the time constants and the knee points, can decrease the mismatch at least in cases of severely impaired hearing at the acoustic ear (Veugen et al., 2016b). Contrary to that, in subjects with moderate hearing loss at the acoustic ear, Spirrov et al. (2020) did not find significant differences between standard and matched AGCs after investigating the effect of matching the compressors. Instead, they suggested that, due to differences in dynamic range between CI and HA, it is necessary to optimize the gain-control step to obtain a similar loudness on both ears (Spirrov et al., 2018). A different take on optimizing the time constants builds on the relation between the ideal compression speed and the patient's short-term memory. Whereas the results of some studies (Leijon, 2017; Ohlenforst et al., 2016) support such an assumption, others (Spirrov et al., 2018) could not identify any influence of short-term memory on bimodal performance.

Overall, level balancing appears to be one of the most important and one of the most difficult fitting aspects for bimodal CI. Much has been done, and much more can be done in the future.

## Clinical Outlook

In the previous sections, we discussed the causes of the interaural mismatches (section “causes of interaural mismatches”), mismatch measurement techniques (section “mismatch measurement techniques”), and compensation strategies (section “mismatch compensation and side effects”) for each of the three dimensions level, latency, and frequency. In section “mismatch compensation and side effects”, we noted how inter-dependent the three dimensions are, and that a large gap remains between knowing these strategies and having a comprehensive and practicable fitting protocol. The goal of the present section is to describe the various aspects of this knowledge gap and to discuss some paths that researchers and audiologists follow or may follow in the future to jointly work towards a clinically feasible bimodal fitting protocol. One focus is to work out the consequences of the interdependencies for the measurement order and compensation order. To limit the complexity and number of different cases, we primarily consider the case that unilaterally optimal fittings of both HA and CI exist and that the patients’ acoustic hearing ear is considered the “better ear”, e.g., with respect to speech understanding. We make the simplifying assumption that in such a case, level and frequency mismatches are best compensated for by altering the CI parameter settings such that the better ear performance is not compromised.

In the same vein as in the previous sections, an ideal facility is envisaged. Practical limitations, such as the availability of imaging equipment or staff expertise to fit both HA and CI, play a central role in how a clinic organizes the fitting routine. Here, we will generally assume a best-case scenario, but are aware of inevitable constraints, such as the available time per fitting session, or patients’ ability and willingness to perform extended listening tasks. We will also point out where the present reality differs or is expected to differ from the best-case scenario. Just as above, this section does not address pediatric fitting or the fitting of patients with severe hearing loss in the acoustically stimulated ear.

The best-case scenario for the fitting procedure of either newly or long-term implanted patients is based on the following assumptions: (1) The electric and acoustic latency up to wave V (including the device latency) of a bimodal or SSD-CI user is already known or confidently estimated from known device latencies (see section “frequency mismatch measurements”). (2) A CT image of the inserted electrode array is available, no electrodes are deactivated and no dead regions are present. (3) It is possible to flexibly change CI stimulation levels and frequency allocation and to increase processing latency at either device in a frequency specific manner (The latter cannot yet be expected in practice). Apart from optimistically assuming that this technology is readily available when these lines are read, we follow the previous sections with typical present-day devices and technology in mind, such as the clinically available speech-coding strategies.

At the end of the fitting procedure, the optimal outcome would be that the spatial hearing performance of bimodal users reaches that of their bilateral CI peers (Deep et al., 2020). The main bimodal and SSD-CI benefits for patients with relatively good acoustic hearing are sound localization, spatial awareness, speaker segregation (e.g., Bernstein et al., 2016), and an improved listening comfort. As speech understanding in quiet and noise is already expected to be relatively good, due to the acoustic hearing, for these patients the “weaker ear” is expected to primarily improve speech understanding in cases where the interference is on the side of the better-hearing ear (Williges et al., 2019). Therefore, the primary goal of the fitting process described in the following is to optimize sound localization and spatial awareness. Speech intelligibility is addressed by - as far as possible - retaining the unilaterally optimized fitting of the better ear. As in the previous sections, there is a subsection for each of the three fitting dimensions. Here, however, a chronological description of the bimodal fitting protocol is implied. Additionally, there is one subsection on interdependencies and one highlighting practical implementation issues.

### Latency Fitting

Latency compensation is an ideal starting point, because it is less affected by the other stimulation parameters or mismatch factors. Evidently, the device on the side with a shorter compound latency should be delayed in a frequency-specific manner (see Table 1). In the case of a shorter latency at the acoustic ear, the HA would require a latency increase, but in hearing aids this option is less likely to be available, which may influence the choice of HA in favor of a device with higher latency. Also, for SSD-CI users, a compensation is not possible with shorter latency at the acoustic ear, so that a short CI latency – or more precisely a short latency of the speech processor – is an argument for the choice of device. At present, latencies differ between CI manufacturers, but not (or only slightly) within a manufacturer's device portfolio, so that this choice would have to be made pre-operatively.

### Frequency Fitting

**Figure 5.**
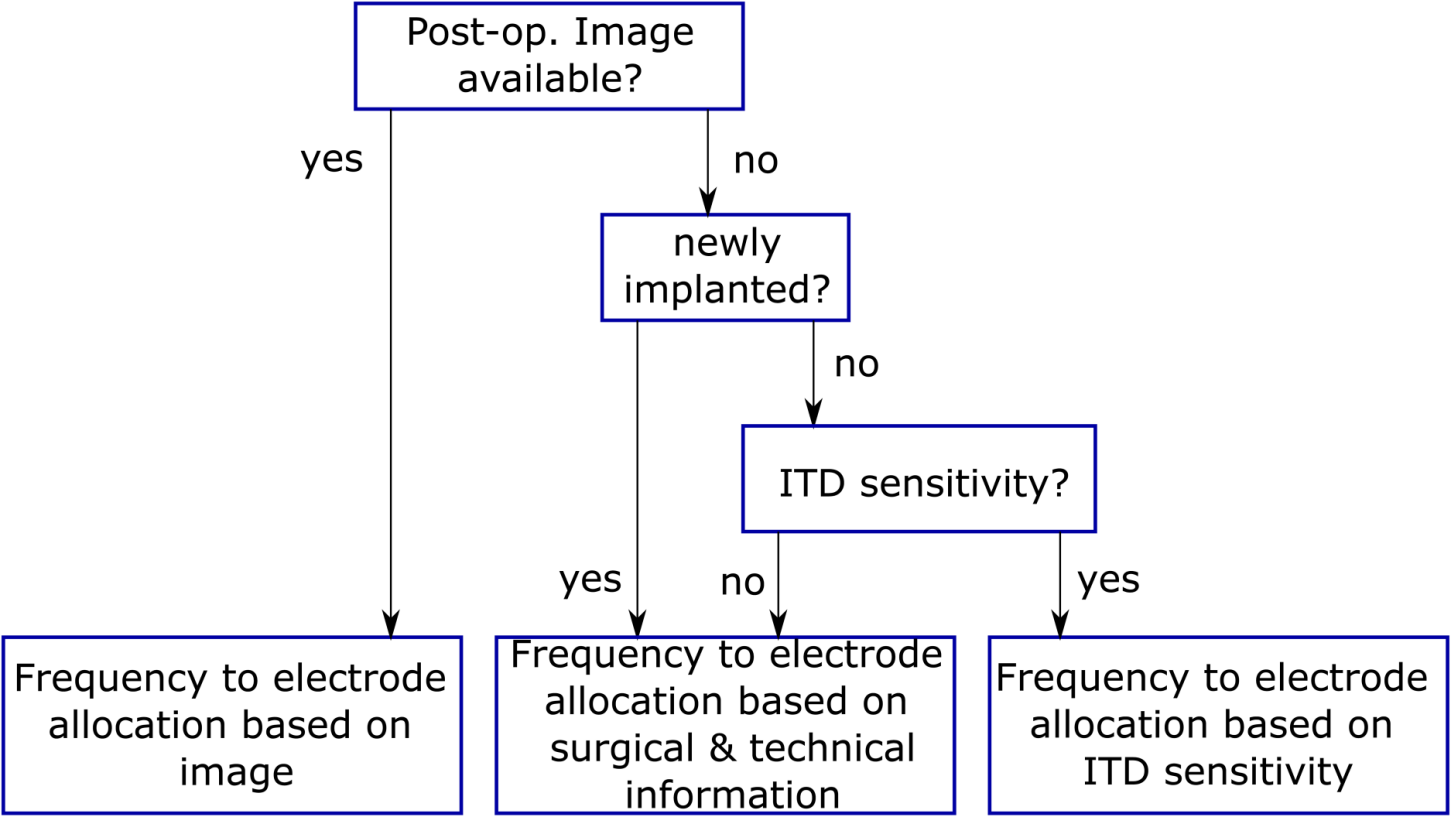
Decision tree to compensate the frequency mismatch between the electric and acoustic ear (top to bottom).

Following the compensation of the latency mismatch, the next step is to reduce the frequency mismatch (Figure 5) by adjusting the frequency allocation table based on the CT/X-ray image (see section “frequency mismatch measurements”).

Apart from our best-case scenario, there might be cases in which no postoperative image of the inserted electrode is available. With pitch matching having several disadvantages and determining the BIC facing several challenges (see section “frequency mismatch measurements”), the measurement of ITD sensitivity along the electrode array might be a good alternative, as well as estimating the corresponding frequency using surgical and technical information (see section “frequency mismatch measurements”). The latter might be an option especially for CI users, that struggle with sensitivity towards ITD. Additionally, it should be noted, that although not all clinics perform post-operative CT scans, in most cases at least a post-operative x-ray image is part of the clinical routine. Therefore, the x-ray can be a good compromise to perform image-based fitting if a good quality of the image can be provided.

### Level Fitting

**Figure 6.**
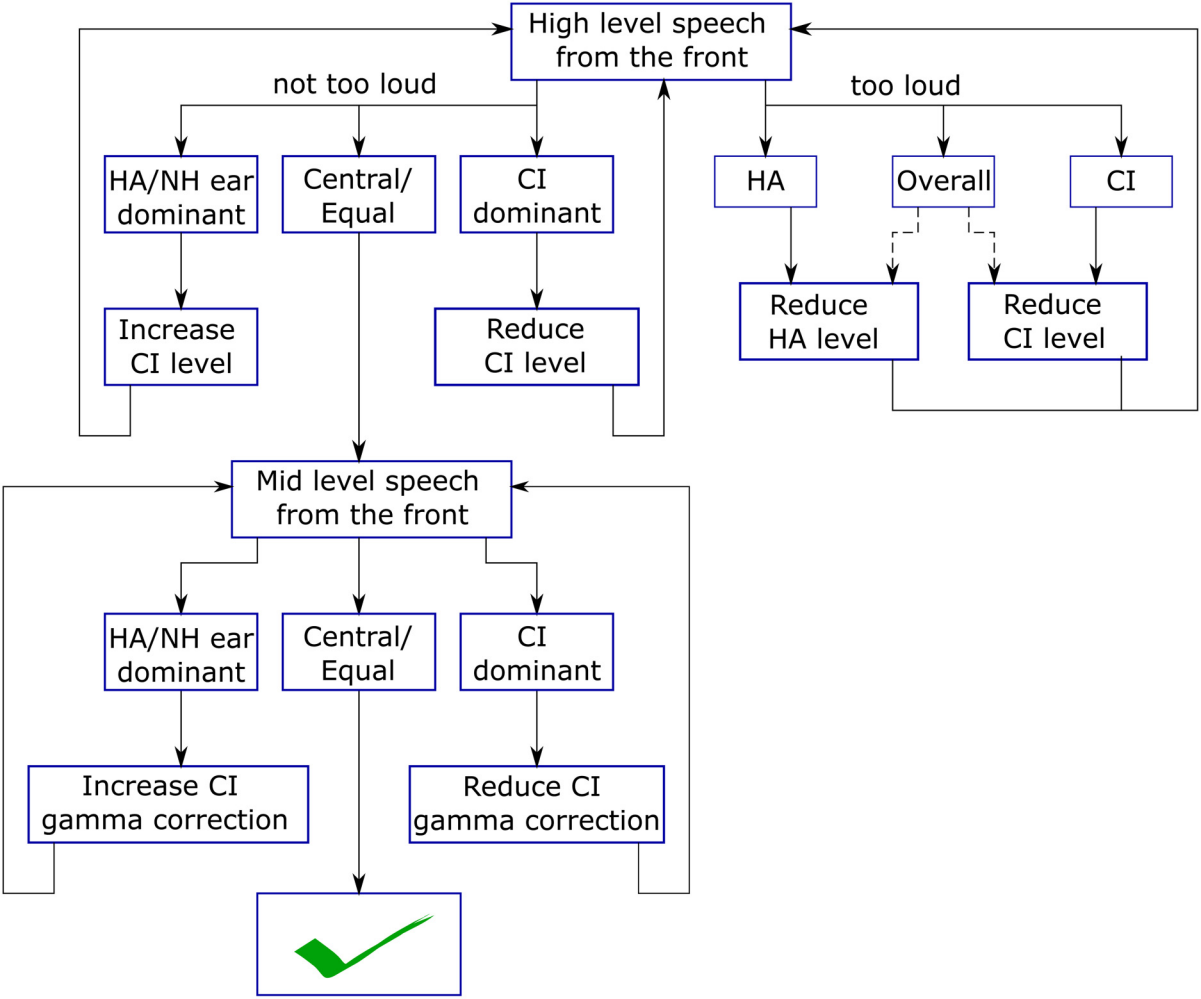
Decision tree to compensate the loudness mismatch between the electric and acoustic ear (top to bottom). Starting with high level speech from the front, a reduction of CI or HA level might be necessary in case of an uncomfortable binaural loudness (right loop). Otherwise the levels are adjusted to receive a central/equal loudness perception (left loop).

Newly implanted CI users do not initially tolerate high stimulation levels, and their sensitivity to level changes considerably over the first few weeks. It is unlikely that a time-consuming precision adjustment is in the interest of either the clinician or the patient, given the fitting's short expected life span. Only after completing the acclimatization phase does it make sense to compensate the level mismatch between the acoustic and the electric ear, and the existing fitting can be used as a starting point. We expect that for the most part, the adjustment will be performed broadband. An example approach is illustrated in Figure 6. In the case of a fused sound image, the adjustment should aim for a centralized perception; otherwise, an equal loudness between left and right is the goal (see section “level mismatch measurements”). For SSD-CI users, the configuration is obviously only possible at the CI, while for bimodal CI users, the best configuration can be obtained when access to the fitting parameters of both devices is possible. However, as mentioned above, the case considered in this draft protocol changes solely the CI parameters.

As a first step, we propose testing whether a frontally presented, high-level stimulus is perceived as too loud. As little is known about binaural loudness in bimodal and SSD-CI users, the possibility exists that the bilateral presentation is far too loud, despite each unilateral stimulation in isolation being acceptably loud (Oetting et al., 2016). This case must be dealt with by uni- or bilateral level reduction, and should be adjusted first. Then, continuing with this high-level broadband signal, the level can be adjusted on the CI side until the perception is balanced (i.e., centralized or equally loud). Once a balance is reached, the same procedure should be repeated with a reduced source level. If the percept is now biased to one side, the nonlinear mapping from input level to stimulation level should be adjusted accordingly. Often this is possible by means of a power-law or gamma correction, e.g., the maplaw parameter in the fitting software for MED-EL devices. If this does not lead to a level-independent, bias-free stimulation, other level-affecting parameters need to be adjusted (e.g., gain-control step or AGC parameters; see section “level mismatch compensation”).

Further fine-tuning of the level parameters is possible by using a more complex approach, with a frequency-specific narrowband stimulation. This approach allows for a more accurate adjustment of the level parameters. However, to reach the necessary accuracy with frequency-specific measurement techniques, long measurement times are necessary, which means that such methods are most likely not time-efficient enough for a clinical setup. Some novel approaches to overcome this inconvenience while preserving the needed accuracy - e.g., improving the efficiency of categorical loudness scaling, are currently being examined (e.g., Fultz et al., 2020). It should, however, be kept in mind that in the narrowband approach, for some bimodal CI a centralized or equal-loudness percept along the entire frequency range might not be possible, e.g., with the HA dominating at low frequencies and the CI dominating at high frequencies (see section “level mismatch compensation”). In these cases, an accurate narrowband tuning of the level parameters is not expected to be possible and should not be the goal. However, within the mid-frequency range, there should be a higher chance of success. A loudness-scaling procedure will give the most detailed insight about the loudness growth of the HA/NH ear and the CI. Time-efficient equalization strategies (e.g., Francart & McDermott, 2012) are necessary if such detailed approaches are to be adopted in a clinical protocol.

### Interdependencies

Although we have suggested the fitting order: 1) latency, 2) frequency, 3) level for our best-case scenario, it may be necessary to alternate between the three fitting dimensions or to iterate through the process for a second time. This is due to the interdependencies between the dimensions. Latency, for instance, which we have argued to be least dependent on the other parameters, nevertheless depends on level in acoustic stimulation, but much less so in electric stimulation (Abbas & Brown, 1988). Similarly, in the case of a given frequency-specific device latency, an adjustment of the frequency allocation will alter the band-specific latency match. Other interdependencies as the interdependency between level and frequency are even more critical and were discussed in section “mismatch compensation and side effects”. Particularly noteworthy is the case where the first round of mismatch compensation improves binaural fusion. As discussed above (see section “causes of level mismatch” and “level mismatch measurements”), binaural fusion may lead to a different level matching (Figure 4) but this aspect of bimodal fitting has not yet been studied.

### Reality Check

In the previous sections (“latency fitting” to “interdependencies”), we assumed a best-case scenario. As the name suggests, this is clearly not a “one fits it all” guideline, but is rather expected to have various practical limitations.

First, the control of one of the devices may be limited either by manufacturer constraints or by staff-specific limitations. An example for the former is that not all CIs allow for a completely custom frequency allocation. An example for the latter is that HA and CI fitting is often performed in a sequential manner by two different professionals. Similarly, whereas each device is typically fitted within its own framework, a combined CI and HA fitting software is arguably the best approach, but only available for a few combinations of partner-brand devices (e.g., Holtmann et al., 2020).

The theory behind bimodal fitting laid out in this and other articles is so complex that even dedicated researchers may not always be able to fully grasp the complex interplay. Concentration on some essential components will be inevitable and typical. At this stage, the suggestions presented here primarily address early adopters, such as research audiologists in large centers. Knowledge translation to the clinical routine is then the second step, but needs to be considered early on (e.g., Moodie et al., 2011). Particularly in the clinical routine, one has to consider that a center may not be able to perform certain measurements (e.g., no CT imaging being part of the clinical protocol or to avoid additional radiation) or find certain other measurements to be too time consuming.

### Outcome Measures

After setting all parameters, it is important to verify if the fitting improves hearing in tests that reflect real-life. For newly implanted patients a comparison towards the preoperative results can be achieved. However, it rather displays the success of a fitting compared to no CI rather than the actual success of the compensation itself. Nevertheless, it might give some insights, if further optimization of the fitting might be necessary and serve as a baseline for longitudinal improvement or future fittings. In contrast, for long-term CI users who may have received their first “binaurally optimized fitting” to compensate for their mismatches, a direct comparison before and after the mismatch compensation is possible and allows a judgement about successful mismatch reduction. To allow comparable outcome measurements among different centers, van de Heyning et al. (2016) worked on a unified test framework for SSD-CI patients, that could also be used for bimodal CI users. This test framework includes speech in noise testing with different spatial configurations. This allows for comparing different binaural benefits such as head-shadow and binaural contrast. In addition, a test for concurrent speaker segregation might be useful, as improving source segregation is one of the major motivations to reduce mismatches. Also, improving binaural fusion due to compensated mismatches is of central importance. Reports on the benefit of latency compensation (section “latency mismatch compensation”) by means of localization accuracy (Angermeier et al., 2021; Zirn et al., 2019) are good examples of outcome measures. They also highlight the relevance of acclimatization, which was fortunately fast in their case, but is possibly longer in case of frequency remapping. Even the most involved laboratory testing may fall short to resemble real life. Inferring from patient reports by means of formal questionnaires is therefore another useful source of information (e.g. van de Heyning et al., 2016). All these attempts towards objective outcome measures notwithstanding, the informal patient report interpreted by an experienced audiologist with some personal knowledge about their patients auditory and non-auditory attributes is certainly required to evaluate what the best possible outcome is for each individual patient.

## Conclusions

The complexity of fitting SSD- and bimodal CI patients is reflected in the length of the present text. Four examples of conclusions distilled from the literature are:

1. A reduction of interaural mismatch in frequency and latency improves binaural fusion. Without binaural fusion the two ears act as two almost independent receivers. With binaural fusion we expect better localization and possibly improved masking release but we have to revisit some concepts such as loudness balancing and anticipate a more involved fitting process.
2. The three dimensions level, latency, and frequency are interdependent.
3. A mismatch in one dimension can obliterate the benefits of matching in other dimensions.
4. Level balancing is not always expected to be possible such that the patient perceives all frontal sources from the front.

This sobering summary is part of the reason why an elaborate bimodal fitting protocol is far from clinical routine. Binaural fusion is critical in formulating the fitting goal, but often not considered. With improving device technology, such as adjustable latency, or a wireless information exchange, and with more bimodal patients with good acoustic hearing, the demand for a smart fitting strategy will increase. Fitting tools are also improving, most notably CT-based imaging, but the task is not expected to get much easier.
